# Therapeutic Antibodies: What Have We Learnt from Targeting CD20 and Where Are We Going?

**DOI:** 10.3389/fimmu.2017.01245

**Published:** 2017-10-04

**Authors:** Michael J. E. Marshall, Richard J. Stopforth, Mark S. Cragg

**Affiliations:** ^1^Antibody and Vaccine Group, Cancer Sciences Unit, Faculty of Medicine, University of Southampton, Southampton General Hospital, Southampton, United Kingdom

**Keywords:** anti-CD20, monoclonal antibody, Fc gamma receptors, Fc engineering, isotype, resistance, combination therapies

## Abstract

Therapeutic monoclonal antibodies (mAbs) have become one of the fastest growing classes of drugs in recent years and are approved for the treatment of a wide range of indications, from cancer to autoimmune disease. Perhaps the best studied target is the pan B-cell marker CD20. Indeed, the first mAb to receive approval by the Food and Drug Administration for use in cancer treatment was the CD20-targeting mAb rituximab (Rituxan^®^). Since its approval for relapsed/refractory non-Hodgkin’s lymphoma in 1997, rituximab has been licensed for use in the treatment of numerous other B-cell malignancies, as well as autoimmune conditions, including rheumatoid arthritis. Despite having a significant impact on the treatment of these patients, the exact mechanisms of action of rituximab remain incompletely understood. Nevertheless, numerous second- and third-generation anti-CD20 mAbs have since been developed using various strategies to enhance specific effector functions thought to be key for efficacy. A plethora of knowledge has been gained during the development and testing of these mAbs, and this knowledge can now be applied to the design of novel mAbs directed to targets beyond CD20. As we enter the “post-rituximab” era, this review will focus on the lessons learned thus far through investigation of anti-CD20 mAb. Also discussed are current and future developments relating to enhanced effector function, such as the ability to form multimers on the target cell surface. These strategies have potential applications not only in oncology but also in the improved treatment of autoimmune disorders and infectious diseases. Finally, potential approaches to overcoming mechanisms of resistance to anti-CD20 therapy are discussed, chiefly involving the combination of anti-CD20 mAbs with various other agents to resensitize patients to treatment.

## Introduction

**Figure 1:**
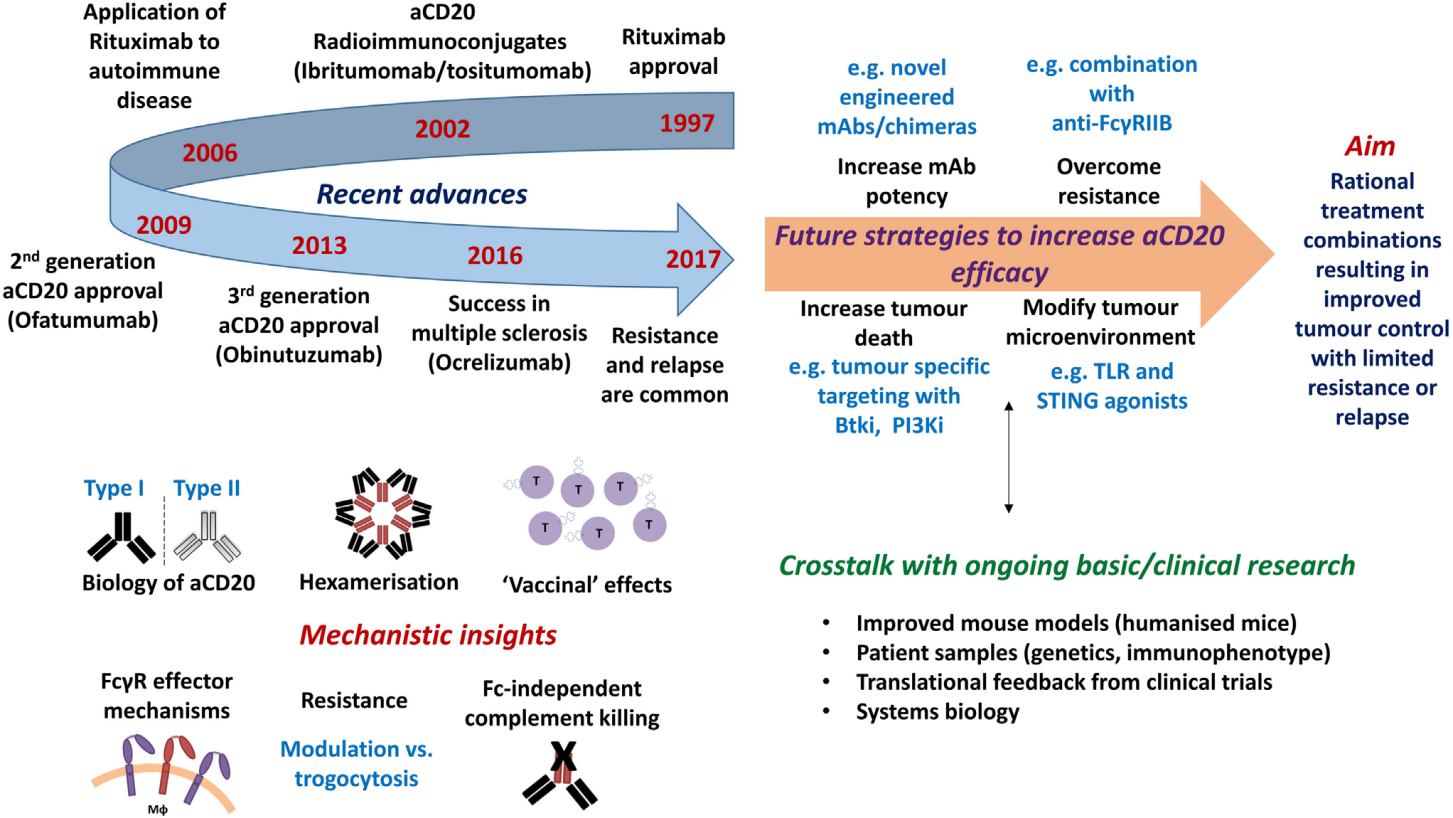
Timeline of approvals and recent discoveries arising from the study of anti-CD20 monoclonal antibodies (mAb), with proposals of how efficacy may be further augmented. Top left: timeline of notable clinical developments of anti-CD20 mAb. Bottom left: recent mechanistic insights gained from the study of anti-CD20 mAb. Top right: future strategies required to increase the efficacy of anti-CD20 mAb. Bottom right: technical developments and knowledge required to further inform therapeutic design.

Over the last two decades, monoclonal antibodies (mAbs) have become a key part of treatment regimens for many diseases, including cancer. In 1997, rituximab became the first mAb to receive Food and Drug Administration (FDA) approval in oncology for relapsed/refractory non-Hodgkin’s lymphoma (NHL), and has since significantly impacted on a vast number of patients with various B-cell malignancies and, more recently, autoimmune disorders (1, 2). For example, addition of rituximab to conventional [CHOP; cyclophosphamide, hydroxydaunorubicin, vincristine (Oncovin), prednisolone] chemotherapy in diffuse large B-cell lymphoma (DLBCL) has resulted in significantly increased progression-free and overall survival at 10-year follow-up (3, 4). By contrast, treatment success is more modest in conditions such as chronic lymphocytic leukemia (CLL) and mantle cell lymphoma (MCL), where response rates are lower and many patients relapse and/or become refractory to treatment (5). Both the success and failure of rituximab has driven the development of further mAb reagents; leading to an increase in our knowledge of how mAb work and how resistance arises (Figure 1).

Interestingly, although much of the current focus in immunotherapy is on checkpoint blockers and other immunomodulatory mAb; in fact, the majority of mAbs approved for use in oncology are the so-called direct-targeting mAb, such as rituximab (6), which are designed to target tumor cells directly. Indeed, mAbs targeting CD20 represent over a quarter of such tumor-targeting mAbs with more in clinical development for conditions outside of cancer (Table 1). Moreover, as many immunomodulatory mAb such as anti-CTLA-4, GITR and OX40 may function as direct-targeting mAb, by deleting regulatory T cells (Tregs) (7–9), the lessons we have learnt from CD20 likely have further relevance in these settings.

**Table 1:** Direct-targeting monoclonal antibodies (mAbs) currently approved for use in oncology settings.

Generic name	Brand name	Target	Format	Comments (anti-CD20)	Indication	Food and Drug Administration (FDA) (EMA) approval date/status	Reference
Rituximab	MabThera; Rituxan	CD20	Chimeric IgG1	Type I	NHL	1998 (1997)	(1–5)
Ibritumomab tiuxetan	Zevalin	CD20	Mouse IgG1	Type II, ^90^Y radiolabeled	NHL	2002 (2004)	(10, 11)
Ofatumumab	Arzerra	CD20	Human IgG1	Type I, binds small CD20 loop	CLL	2009 (2009)	(12, 13)
Obinutuzumab	Gazvya; Gazyvaro	CD20	Humanized IgG1	Type II, glycomodified	CLL	2013 (2014)	(14–17)
Ocrelizumab^a^	Ocrevus	CD20	Humanized hIgG1	Type I	MS	2017 FDA (under review by EMA)	(18, 19)
Veltuzumab^a^	N/A	CD20	Humanized hIgG1	Type I, rituximab backbone	Various (i.e., NHL; CLL; ITP)	Clinical trials and/or FDA orphan drug status	(20, 21)
Ocaratuzumab^a^	N/A	CD20	Humanized hIgG1	Type I, Fc-modified	FL; CLL	As above	(22, 23)
Ublituximab^a^	N/A	CD20	Chimeric hIgG1	Type I, glycoengineered	Various (i.e., CLL; MS; other)	As above	(24, 25)
Cetuximab	Erbitux	EGFR	Chimeric IgG1		Colorectal cancer	2004 (2004)	(26)
Panitumumab	Vectibix	EGFR	Human IgG2		Colorectal cancer	2006 (2007)	(27, 28)
Necitumumab	Portrazza	EGFR	Human IgG1		NSCLC	2015 (2015)	(29)
Trastuzumab	Herceptin	HER2	Humanized IgG1		Breast cancer	1998 (2000)	(30, 31)
Pertuzumab	Perjeta	HER2	Humanized IgG1		Breast cancer	2012 (2013)	(32, 33)
Ado-trastuzumab emtansine	Kadcyla	HER2	Humanized IgG1	Drug conjugate	Breast cancer	2013 (2013)	(34, 35)
Brentuximab vedotin	Adcetris	CD30	Chimeric IgG1	Drug conjugate	NHL; large cell lymphoma	2011 (2012)	(36, 37)
Daratumumab	Darzalex	CD38	Human IgG1		Multiple myeloma	2015 (2016)	(38, 39)
Dinutuximab	Unituxin	GD2	Chimeric IgG1		Neuroblastoma	2015 (2015)	(40, 41)
Alemtuzumab	Lemtrada, MabCampath	CD52	Humanized IgG1		CLL; MS	As Campath—2001 (2001)	(42, 43)
						As Lemtrada—2014 (2013)	
Olaratumab	Lartruvo	PDGFRα	Human IgG1		Soft tissue sarcoma	2016 (2016)	(44, 45)

*Table modified from (46)*.

*^a^Additional anti-CD20 mAbs in clinical development and/or for clinical indications outside of cancer are also shown. Withdrawn mAbs are excluded*.

*NHL, non-Hodgkin’s lymphoma; CLL, chronic lymphocytic leukemia; MS, multiple sclerosis; ITP, idiopathic thrombocytopenic purpura; FL, follicular lymphoma; NSCLC, non-small cell lung cancer; EGFR, epidermal growth factor receptor; HER2, human epidermal growth factor receptor 2; GD2, disialoganglioside 2; PDGFRα, platelet-derived growth factor receptor alpha*.

In this article, we review developments arising from targeting CD20 and then discuss a range of approaches that are now being applied to improve efficacy, including new antibodies and combination strategies.

## CD20 as a Model Target

The pan B-cell marker CD20 remains one of the best studied antibody targets to date. Originally named B1, CD20 was discovered in 1980 as the first specific B-cell marker (47). It is a non-glycosylated tetraspanin of the membrane spanning 4-A family, with two extracellular loops (48–50) containing the epitopes for anti-CD20 antibodies (51).

Early studies showed that CD20 forms homotetramers in the cell membrane, suggesting that it may function as an ion channel, and that it disassociates from the B-cell receptor (BCR) upon mAb binding (52). CD20 is now thought to modulate calcium release arising from the BCR: CD20-deficient mouse cells exhibit decreased calcium signaling downstream of BCR engagement, and human B-cells (Ramos) are unable to initiate calcium signaling in the absence of the BCR despite CD20 crosslinking (53, 54). In mice and humans, loss of CD20 results in defects in the ability to generate antibody responses to certain antigens (55, 56).

Importantly, as well as being expressed on normal B-cells, CD20 was also found to be expressed on the surface of malignant B-cells (57). Furthermore, CD20 is expressed on pre-B-cells from an early stage in their development but is not present on the precursor hematopoietic stem cells from which they are derived, and expression is lost during differentiation into antibody secreting plasma cells (58–60). This expression pattern is close to ideal for a target antigen: it minimizes the potential for off-target toxicity and retains humoral protection against previously encountered pathogens (61), while allowing for repopulation of the B-cell compartment after cessation of anti-CD20 treatment.

Another property that affords CD20 ideal target antigen status is its expression level: it is highly expressed, with approximately 100,000 CD20 molecules expressed on the surface of normal B-cells (with similarly high levels on most malignant cells) (62), which facilitates efficient target opsonization and deletion (63). Moreover, given the extracellular structure of the molecule, the available mAb binding epitopes are located close to the plasma membrane, a feature that has been reported to facilitate efficient binding and recruitment of effector mechanisms for deletion (64, 65). Perhaps less important but also worthy of consideration are that CD20 has no known ligand to interfere with mAb binding and does not exhibit extracellular post-translational modifications, reducing the variation in, and potential loss of, binding epitopes (49).

## Type I and Type II Anti-CD20 Antibodies

Anti-CD20 mAbs also have the capacity to redistribute CD20 within the plasma membrane into lipid rafts (66). Functionally, this redistribution may be important for the role of CD20 in BCR signaling (67). However, it also has significant implications for anti-CD20 antibodies themselves. The ability (or lack thereof) of mAbs to redistribute CD20 into lipid rafts has served as a useful classification system for anti-CD20 antibodies (68, 69). mAbs such as rituximab and ofatumumab that bind CD20 and cause compartmentalization into lipid rafts are classified as type I antibodies, whereas those that bind CD20 but cause no redistribution, such as obinutuzumab, are known as type II antibodies (51). In addition to a convenient basis for antibody nomenclature, the type I/II distinction describes key differences in antibody characteristics: first, opsonization of CD20^+^ target cells with type I mAb results in binding twice as many antibody molecules per cell as a type II antibody (63). This is thought to be due to differences in the modes of binding between the two antibody types, as suggested by X-ray crystallography structures and tomography analysis of type I and II mAbs in complex with CD20 (70). Type I antibodies are proposed to bind CD20 tetramers in a manner that does not block binding of subsequent antibodies, whereas type II antibodies are thought to bind across the tetramer, blocking the binding of further mAbs (51).

The redistribution of CD20 and the associated mAb into lipid rafts is also functionally important with regard to the antibody effector functions induced. Due to the enhanced clustering of antibody Fc regions, type I antibodies are able to potently induce complement-dependent cytotoxicity (CDC), whereas type II antibodies do not induce CDC to a similar extent (51). However, type II antibodies have been reported to induce a greater degree of directly induced, non-apoptotic cell death upon binding to target cells (71). This mechanism has been shown in both B-cell lines as well as primary B-CLL cells (72). The enhanced clustering of type I antibodies renders them more susceptible to internalization, resulting in lysosomal degradation and a reduction in surface CD20 expression (73). Known as antigenic modulation, this is thought to be an important mechanism of resistance to type I anti-CD20 treatment.

Importantly, since the very first studies on CD20 mAb carried out with B1 and 1F5 (74), it has been clear that targeting the same surface marker with different mAb can have profound differences in response. Among many other lessons, this has been an important one that study of CD20 has revealed. In fact, subsequent work by Niederfellner et al. revealed that type I and II mAbs bind an extremely similar epitope on the same loop of CD20 and it is likely that only the orientation of binding differs between these mAbs but that this results in profound differences in activity (75).

## Mechanisms of Direct-Targeting mAb Function

As alluded to above, therapeutic mAbs are able to elicit multiple effector functions after binding to their target antigen. The study of anti-CD20 mAbs has contributed to the understanding of almost all of these, including signaling through the target molecule, triggering cell death, initiating the complement cascade, and engagement of Fc gamma receptors (FcγRs) triggering FcγR-dependent responses such as target cell lysis or engulfment (76).

### Direct Binding Effects

Monoclonal antibody binding can have multiple direct effects on the target cell. For example, binding to a receptor can block binding of the relevant ligand, such as is the case with cetuximab binding the epidermal growth factor receptor (EGFR), inhibiting soluble EGF binding; thereby reducing proliferation and survival signaling to the tumor (26). With CD20, direct effects are again dependent on the mAb type; type I mAb triggering a limited degree of apoptosis, which is likely reflective of BCR signaling and type II mAb provoking a non-apoptotic lysosomal form of cell death (69). How this is triggered is still the subject of much debate, but is likely related to reactive oxygen species production (77).

### Complement-Dependent Cytotoxicity

All anti-CD20 mAb used in the clinic to date have been of the IgG1 subclass and so are able to activate the complement cascade once bound to target-expressing cells, triggering complement-dependent cytotoxicity (CDC). This process begins with the binding of C1q and follows the sequential activation of several proteases that cleave serum complement proteins in a specific order, generating enzymatic complexes that trigger further protein recruitment and processing (78). The end result of the cascade is threefold: the liberation of soluble molecules that act as anaphylatoxins to recruit immune effector cells; the deposition of cell bound cleavage fragments, largely C3b, acting as opsonins promoting target cell phagocytosis; and, finally, formation of a membrane attack complex (MAC) in the target cell membrane (79).

It has recently been shown how the proximity of binding to the membrane affects the effector functions engaged by an antibody, as had been previously suggested by the enhanced complement activating ability of ofatumumab (65, 80). Ofatumumab (2F2) is a type I anti-CD20 mAb (Table 1) that recognizes an epitope comprising both extracellular loops, binding closer to the cell membrane than rituximab (12). This membrane proximity is linked to the increased CDC seen with this antibody compared to rituximab (13). Ofatumumab has shown activity against rituximab-resistant CLL cells *in vitro*, despite their low CD20 expression, and has been approved for CLL treatment (13, 80).

Although CDC has been studied for many years, it was only recently revealed, using mAbs to CD20 and other targets, that IgG adopts a hexameric conformation in order to interact efficiently with the six head domains of C1q (81). The formation of hexamers on the target cell surface results from non-covalent interactions between adjacent Fc regions, increasing C1q binding avidity and subsequent CDC efficacy (81). This observation prompted a series of new developments in mAb engineering. Specific mutations capable of enhancing hexamerization of IgG and hence CDC were identified, namely E345R, E430G, and S440Y (81). Introducing the E345R mutation into anti-CD20 (IgG1-7D8) significantly increased Daudi cell lysis in comparison to wild-type IgG1 (81). In a further study, de Jong et al. showed the applicability of these findings to mAbs targeting different target antigens (i.e., CD52), target cell lines with differing levels of CD20 and complement regulatory proteins, and also confirmed improved efficacy in comparison to wild-type mAb in a tumor model (82).

Despite the obvious potential of such Fc region engineering for enhanced CDC, introducing multiple hexamer-enhancing mutations is likely to be detrimental, as double (E345R/E430G; RG) (82) and triple (E345R/E430G/S440Y; RGY) (81, 82) mutants formed hexamers in solution (RG—7.7%, RGY—73%) (82). RGY also activated complement in the absence of target cells, as measured by C4d generation (81). Although to a lesser degree than double and triple mutants, some single mutants also resulted in the formation of a small percentage of hexamers in solution (1.2% for E345R), target-independent complement activation, and accelerated clearance of antibody from the circulation (82). However, an important finding was that amino acid substitutions at positions E345 and E430 (resulting in enhanced hexamer formation on the target cell) was not restricted to R and G, respectively. Moreover, when the preferred mutations (E435K or E430G) were introduced into the type I anti-CD20 mAbs 7D8 and rituximab, an increase in CDC in 5/6 CLL samples in comparison to wild-type mAbs was observed (with one of the CLL samples being refractory to CDC due to having a very low CD20 expression).

Intriguingly, it was also shown that the inefficient CDC induced by type II anti-CD20 mAbs (11B8) (82), or an anti-CD38 mAb containing IgG2 and IgG4 Fc regions (81) could be partially overcome by introduction of hexamer-enhancing mutations. Alternatively, the poor CDC mediated by anti-EGFR (2F8) was overcome by forcing monovalent binding of antibody to the target (81), indicating that the orientation of mAbs on the target cell is important for hexamer formation. However, CDC mediated by the type I anti-CD20 mAb 7D8 was not enhanced when only capable of monovalent binding (81). Although rituximab is able to adopt a monovalent binding to target antigens due to a relatively high off-rate (83), this explanation for enhanced CDC in the case of 7D8 is unlikely as 7D8 has a lower off-rate (83) and also induces more CDC in comparison to rituximab in the presence or absence of hexamer-enhancing mutations (82). Nevertheless, these results suggest that the CDC capability of a mAb may be increased by forcing hexamerization at the level of the target, and that a single hexamer-enhancing mutation is probably sufficient. However, what remains to be seen is whether these mutations also augment FcγR-mediated mechanisms and elicit greater efficacy *in vivo*.

### FcγR-Mediated Mechanisms

Unique to IgG antibodies are the effects mediated through the FcγR family. These receptors are expressed on many different cell types and are essential for several IgG functions (84). Conventionally, FcγR-expressing effector cell functions have been ascribed to either natural killer (NK) cells or myeloid effectors (85). NK cells are able to mediate a direct lytic attack on opsonized target-expressing cells through FcγRIIIA [and, if present, FcγRIIC (86)] through a process termed antibody-dependent cell-mediated cytotoxicity (ADCC) (87).

Another FcγR-dependent mechanism is mediated by phagocytic cells, such as macrophages, monocytes, and neutrophils. Similar to ADCC, opsonized target cells trigger signaling through FcγRs expressed on the phagocyte, resulting in actin rearrangement and extension of the phagocytic cell membrane (88). The membrane eventually engulfs the opsonized cell in a phagocytic vesicle, or phagosome, which then fuses with lysosomes within the phagocyte, resulting in degradation of the phagocytosed cell by lysosomal enzymes (85). This mechanism has been termed antibody-dependent cell-mediated phagocytosis (ADCP). In fact, myeloid cells can elicit both phagocytosis and killing of targets (89).

## *In Vivo* Mechanisms of Action

The above described effector functions of IgG can all be readily demonstrated through *in vitro* assays (14, 65). However, knowledge of the relative importance of these effector functions to *in vivo* efficacy is essential to design optimal treatments.

One method applied to shed light on *in vivo* antibody function has been the retrospective analysis of the impact of FcγR polymorphisms in human clinical trials. In some trials, this analysis has revealed a significant correlation between the FcγRIIIA V158 polymorphism that encodes for higher affinity binding to IgG1 and clinical response (90, 91). This finding supported the paradigm that FcγR-mediated effector functions and particularly ADCC through NK cells, which predominantly express only FcγRIIIA, were the dominant effector mechanisms for anti-CD20 mAb. These findings also reinforced the bias that NK cells are the principle effectors for anti-CD20 mAb which derives from studies of human peripheral blood mononuclear cells (PBMCs) and blood (in which key effectors such as macrophages and/or neutrophils are lacking). However, it is important to note that several myeloid cells, including macrophages, also express FcγRIIIA and that, more recent, larger oncology trials have failed to show strong evidence for this receptor polymorphism as being central to antibody efficacy (92, 93).

With regard to other effector functions studied in humans, data from samples collected from patients treated with rituximab convincingly show that components of the complement system are depleted after mAb administration, and that supplementation of blood from these patients with additional complement components restores complement-mediated lysis *ex vivo* (94). Furthermore, early studies with rituximab suggested that the expression of complement defense molecules, including CD55 and CD59, on target cells was a predictor of poor response to anti-CD20 treatment (95). However, these studies have not been confirmed (96) and, moreover, several negative associations of complement engagement and mAb effector function have been provided (97, 98). Moreover, a polymorphism in the gene encoding C1qA (A276G), known to influence C1q levels, has been linked to responses to anti-CD20, with FL patients having an AG or AA genotype (lower C1q expression) experiencing a significantly longer time to progression following an initial response to rituximab (99), and patients with DLBCL harboring the AA genotype displaying significantly longer overall survival following R-CHOP (100). This seemingly suggests a detrimental role for complement.

Perhaps the best current models for elucidating *in vivo* effector function are mouse models, which facilitate the manipulation of various effector components to establish their relative contribution to antibody efficacy. Initial studies using mice that are defective in the FcRγ chain, and therefore do not express any activatory FcγR, showed no response to anti-CD20 therapy, indicating that activatory FcγRs are absolutely required for anti-CD20 therapy (101, 102). Similar studies in mice lacking the key complement mediators C1 or C3 have argued against a major *in vivo* role for complement as an effector mechanism of anti-CD20 antibodies (73, 103, 104). Thus, it would appear that FcγR-dependent mechanisms predominate in mediating anti-CD20 therapy in mice.

Studies in mice trying to identify the key cell type(s) for mAb-mediated anti-CD20 depletion have indicated that NK cells are not essential for antibody therapy, as anti-CD20 therapy was effective in mouse strains with defective NK cells or after NK cell depletion (103, 105). Intriguingly, in the study by Uchida et al., mice deficient in perforin, one of the main NK cell effector molecules, were still capable of depleting the majority of circulating/splenic B-cells (103) further supporting the absence of a role for NK cells and ADCC as an effector function in anti-CD20-mediated depletion. However, macrophage depletion using clodronate liposomes resulted in impaired deletion of normal and malignant B-cells during anti-CD20 therapy (73, 103, 104). This finding argues that myeloid cells, and particularly macrophages, are the most important cell type for anti-CD20 therapy, at least in mice. Other evidence for this comes from intravital imaging, in which macrophages within the liver (Kupffer cells) were imaged engulfing opsonized B-cells after anti-CD20 therapy (106). As above, clodronate liposomes completely abrogated anti-CD20 mediated B-cell depletion.

Finally, although the evidence for a role of FcγRs and macrophages in the setting of anti-CD20 is unequivocal, a recent study by Lee et al. (107) indicates that next-generation mAb formats may be able to elicit alternative means of activity. Those authors used a library screening approach to select variants of rituximab with enhanced C1q binding but no FcγR binding, and provided evidence that these mAbs can elicit complement-dependent cellular cytotoxicity (CDCC) and complement-dependent cellular phagocytosis (CDCP) in the presence of serum. In comparison to wild-type rituximab, the aglycosylated variant (RA801) with two complement-enhancing mutations (K320E and Q386R) displayed some activity in FcγR-null mice (107) and is, therefore, worthy of consideration as a novel therapeutic; although it should be noted that the models chosen for study represent cell line tumors which may display little complement defense. As such, further experiments are required in fully syngeneic models targeting normal or malignant B-cells in a more physiological setting to confirm these findings, but nonetheless it represents an interesting approach in settings where FcγR-mediated effector functions may be limited.

## Neutrophils as Alternative Effectors

As described above, macrophages are now widely recognized as key mediators of ADCC/ADCP of IgG-opsonized tumor cells *in vivo*, particularly with regard to anti-CD20 mAb. However, there have also been recent reports that neutrophils may also be involved or at least capable of effector activity with these reagents. Neutrophils are characterized by expression of the glycosylphosphatidyl inositol (GPI)-linked FcγR, FcγRIIIB (CD16B), and to a lesser extent FcγRIIA (108) and, therefore, may be expected to be activated by IgG-opsonized tumor cells. Given their abundance in the circulation, it is reasonable to suggest that they can elicit robust effector function.

It has long been known that IgG mAbs are capable of inducing neutrophil-mediated cytotoxicity against B-cell targets. For example, although dependent on the target cell line, anti-human leukocyte antigen (HLA) class II IgG mAbs were shown to mediate ADCC by neutrophil effectors with a clear hierarchy of isotype (IgG1 > 2 > 3 > 4) albeit less than IgA mAbs (109) (see below). Moreover, in the setting of anti-CD20 mAbs, Golay et al. more recently showed that anti-CD20 IgG mAbs are capable of activating neutrophils and inducing tumor cell phagocytosis, at least *in vitro* (15). Consistent with the neutrophil FcγR expression profile, phagocytosis mediated by a glycoengineered variant of rituximab was blocked with F(ab) fragments of either anti-FcγRIII or FcγRII, and to a greater extent with a combination of both. Intriguingly, as for FcγRIIIA, the highly homologous FcγRIIIB was shown to bind with a higher affinity to afucosylated mAbs in comparison to non-glycomodified mAbs (15). In line with this, neutrophil activation (CD11b upregulation, CD62L downregulation, and cytokine secretion) was greater with the glycoengineered (afucosylated) type II anti-CD20 obinutuzumab in comparison to wild-type rituximab. However, comparisons with a non-glycomodified obinutuzumab were not performed in this setting and so the enhanced activation could not be ascribed solely to tighter binding to FcγRIIIB due to afucosylation. Neutrophils were also clearly capable of mediating cytotoxicity of rituximab-opsonized Raji and Ramos cells in a recent study, with an EC_50_ only slightly higher than with PBMC effectors (107). This was shown to be FcγR dependent, as complement-enhanced, Fc-deficient variants of rituximab (RA801 and RA802) were inefficient in neutrophil-mediated lysis (107). However, these rituximab mutants had restored activity in the presence of neutrophils and serum lacking C9 (so as not to activate MAC formation and classical CDC), with lower EC_50_’s in comparison to wild-type rituximab, which was blocked by mAbs to the complement receptors (CR) 3 and 4 (107). This shows that in addition to ADCC *via* FcγRs, neutrophils can also participate in CDCC of anti-CD20-opsonized targets *via* CRs.

An alternative effector mechanism of neutrophils was recently proposed by Nakagawa et al., whereby target cell apoptosis is triggered through neutrophil-mediated crosslinking of surface bound rituximab (110). Blocking studies and use of afucosylated rituximab variants suggested that FcγRIIIB was responsible for such crosslinking. Intriguingly, this phenomenon mirrors the FcγR-mediated crosslinking reported for pro-apoptotic anti-TNF-related apoptosis-inducing ligand (TRAIL) mAbs (111). Although neutrophil-mediated ADCC mediated by IgG mAbs, such as in the context of anti-EGFR IgG1 and IgG2 (112), anti-HLA class II (109), or indeed anti-CD20 (107) has been reported, neutrophil-mediated ADCC was not observed in this study (110). This possibly reflects a difference between methods of neutrophil isolation or target cells used. Similarly, no neutrophil activation was observed (as measured by upregulation of CD63 and FcγRI), which is possibly related to the fact that FcγRIIIB is GPI-anchored (without an intrinsic cytoplasmic domain) and, thus, is not expected to signal when crosslinked alone (unless through the crosslinking of associated lipid raft-resident kinases). Nevertheless, this mirrors previous findings whereby the crosslinking of pro-apoptotic anti-Fas (113) or agonistic anti-CD40 mAbs (114, 115) did not require intracellular immunoreceptor tyrosine-based inhibitory motif (ITIM)-containing signaling domains of FcγRIIB. Similarly, although effector functions such as ADCC are clearly dependent on the immunoreceptor tyrosine-based activation motif (ITAM) signaling domains of activatory FcγR (116), FcγR-mediated antigen internalization and presentation to T cells is seemingly ITAM-independent (117). This indicates that both activatory and inhibitory FcγR may function independently of their respective ITAM or ITIM domains.

In addition to this *in vitro* work, *in vivo* evidence for a role of neutrophils in the killing of IgG mAb-opsonized tumor cells has also been provided. Although not in the setting of anti-CD20, neutrophils protected against tumor growth following IgG mAb therapy in subcutaneous solid tumor models (melanoma and breast cancer), in an FcγR-dependent fashion (118). However, the model used (solid tumor versus hematological) is important to consider, and utilizing the same conditional neutrophil-depletion strategy in B-cell models involving anti-CD20 treatment would be worthwhile. Indeed, in our own studies, depletion studies showed that anti-CD20 mAb-mediated B-cell depletion was independent of neutrophils (119).

Despite the above findings, neutrophil-mediated phagocytosis following mAb engagement is contentious, as a recent study indicated that neutrophils instead mediate the removal of mAb/CD20 complexes from the target cell, in the absence of phagocytosis or target cell death, in a mechanism known as trogocytosis (120). This activity would be expected to be of detriment to the success of mAb therapy. Surprisingly, this trogocytosis was greater for rituximab in comparison to obinutuzumab. In addition to our work on CD20 modulation (73, 121), this may provide a further/alternative explanation for the improved efficacy of obinutuzumab over rituximab observed in CLL patients (16). Similarly, neutrophils have abundant pro-tumor properties (122), suggesting that recruiting neutrophils by direct-targeting mAbs may be undesirable for clinical outcomes.

In summary, IgG mAbs are clearly capable of activating neutrophils. However, potential detrimental functions (i.e., trogocytosis; pro-tumoural functions) should be considered, and the precise role of neutrophils downstream of IgG mAb therapy requires clarification in further studies. Finally, as discussed below, IgG may not be the optimal isotype for recruitment of the favorable attributes of neutrophils, such as ADCC and cytokine/chemokine release (123).

## Vaccinal Responses to mAb Therapy

The principle success of anti-CD20 mAb has been the direct deletion of the target cells by the effector mechanisms detailed above. However, deletion of tumor cells and their engulfment by myeloid effectors raises the possibility of the induction of a T-cell-mediated immune response to the foreign (mutated) components of the tumor. Although this concept has existed for several years, strong evidence in humans has not been forthcoming with the possible exception of data showing the *ex vivo* re-stimulation of T cells from a small number of patients post-rituximab therapy (124). Regardless, ascribing this activity to mAb-mediated killing of the tumor following FcγR-mediated uptake has not been possible. For this reason, more mechanistic proof of concept has been attempted in mouse models.

Dendritic cells (DCs), *via* their surface FcγRs, are adept at internalizing, processing, and presenting or cross-presenting antigen (Ag) to CD4^+^ and CD8^+^ T cells *in vivo*, as highlighted in recent experiments whereby Ag was targeted to specific FcγRs (117). In relation to tumors, however, early work showed that DCs, when loaded with immune complex (IC) and transferred into mice, are capable of presenting Ag to T cells and inducing immune responses that lead to tumor elimination in an antigen-specific manner (125). It was also indicated that FcγRIIB regulates DC maturation in response to IC and, therefore, the magnitude of anti-tumor T cell responses *in vivo* (126). This was expected based on previous studies showing that FcγRIIB regulates the activity of ICs in *in vivo* alveolitis models (127).

An advance came from studies indicating that such T cell responses will develop *in vivo* following anti-CD20 mAb therapy, rather than *via* artificially generated ICs. First, in a series of tumor challenge and rechallenge experiments, Abes et al. showed that when treated with an anti-CD20 mAb, mice were resistant to tumor growth on rechallenge, and this was dependent on the mAb Fc region (128). Recently, the FcγR and cellular requirements for such adaptive, vaccinal effects of mAb therapy using the same model were identified. Using a series of experiments involving conditional DC knockouts, Fc-modified mAbs, and humanized mice, DiLillo and Ravetch provided indirect evidence that macrophage ADCC (*via* FcγRIIIA), DC uptake of ICs (*via* FcγRIIA), and Ag presentation were responsible for the induction of anti-tumor adaptive responses (129).

Intriguingly, both these studies indicated the generation of an adaptive response specific for the CD20 antigen itself, as evidenced by poor survival of mice rechallenged with tumors lacking CD20 (128, 129). Although there are various limitations with these models, such as the utilization of a xenoantigen (human CD20) in mouse (EL4) cell lines, a more recent study also showed that T cells were required for tumor regression of murine A20 tumors following anti-CD20 therapy, as no tumor regression was observed in nude (T cell deficient) mice (130). Notably, Ren et al. also showed a similar requirement for both macrophages [*via* production of type I interferon (IFN)] and DCs in the induction of anti-tumor T cell responses following anti-CD20 therapy, and that CTLA-4^hi^ Treg cells, within larger (more established) tumors, may be responsible for “adaptive resistance.” This lends support for an anti-CD20/anti-CTLA-4 combination regimen. However, the particular tumor model employed is likely important, as the anti-CD20/CTLA-4 combination is not effective in all models (unpublished data).

Despite being slightly different in their T cell subset requirement, with CD4^+^ (128) versus CD8^+^ T cells (130) being more important for primary tumor clearance following anti-CD20 mAb therapy, the mechanisms involved in the various models are not necessarily mutually exclusive. Specifically, IC formation following initial ADCC, which are then internalized/endocytosed and presented/cross-presented by DCs, likely remains the common link. Similarly, the indicated requirement for macrophage type I IFN may help to explain the efficacy of stimulator of interferon genes (STING) agonist/anti-CD20 combination in our own experiments (119). Furthermore, considering the regulatory role of FcγRIIB at the level of the DC, it can be hypothesized that anti-FcγRIIB mAbs in combination with anti-CD20 mAbs (131) (clinical trial NCT02933320, see below) may favor enhanced activation of DCs by ICs following ADCC, migration to lymph nodes and stimulation of anti-tumor T cells.

Finally, this phenomenon is likely not limited to anti-CD20 mAbs, as similar observations were made using an anti-human EGFR2 (HER2) mouse model (132). In summary, in addition to the principle 4 mechanisms (direct effects, CDC, ADCC, and ADCP), the vaccinal effect of mAb therapy is emerging as an additional potential mechanism of action for direct-targeting mAbs. The above studies did not measure IC production *per se*. It is, therefore, of interest to determine how changes in the nature of ICs (size/valency) influence the vaccinal response (i.e., between different patients, cancer types and treatments, etc.). Recent studies have attempted to define the relationship between various IC parameters and FcγR binding and activation (133), and novel assays for the detection of ICs in serum may also assist this endeavor.

## Enhancing Anti-CD20 mAb Function through Fc Engineering

With the progress outlined above in identifying *in vivo* mechanisms of anti-CD20 antibody therapy and the importance of activatory FcγRs, second- and third-generation anti-CD20 antibodies have been developed which utilize several strategies to try and achieve greater efficacy (Figure 1; Table 1).

### Glycoengineering

Removal of the Fc glycans results in a dramatic decrease in binding to FcγRs and complement activation without affecting antigen binding (134–136). This is thought to be due to changes in the constant heavy (CH) 2 domain structure, possibly through the two CH2 domains collapsing to block the FcγR/C1q binding site (137). However, the importance of Fc glycosylation extends beyond simply holding the Fc structure in place (138). Shields et al. found that removal of the core fucose residue, present on most recombinant and serum IgG molecules, resulted in increased FcγRIIIA binding up to 50 times, translating into increased NK-mediated ADCC (138). Shinkawa et al. confirmed this and reported increased ADCC using low fucose anti-CD20 mAb (139).

In 2006, the structural basis for this increased binding was reported, with Ferrara et al., showing *via* X-ray crystallography that the fucose residue was sterically blocking a stacking interaction between the Fc glycans and those present on the Asn162-linked glycan of FcγRIIIA (140). Absence of the fucose resulted in a closer interaction, explaining the increased affinity. As a result of these findings, several afucosylated antibodies have been developed which exhibit the expected increase in FcγRIIIA affinity and ADCC. Currently, afucosylated mAbs targeting CD20 (obinutuzumab) or CC chemokine receptor 4 (CCR4) (mogamulizumab) produced *via* cell line engineering have been brought to the clinic and more may follow (141). While other glycoforms have been linked to specific functions, none have been carried forward to the clinic.

Additional glycomodified anti-CD20 mAbs have been developed, further to obinutuzumab. EMAB-6, an afucosylated anti-CD20 mAb was generated with a view that it may allow lower doses of chemotherapy used in the treatment of CLL (142). This mAb was able to both bind FcγRIIIA more tightly and mediate greater NK-mediated ADCC of CLL cells at lower mAb concentrations in comparison to rituximab (142). A later version of this mAb (LFB-R603, now known as ublituximab) was able to elicit maximal ADCC of target Raji cells at a concentration of 1 ng/ml, in comparison to 100 ng/ml for rituximab (143). Moreover, ublituximab recently showed promising efficacy when combined with the Bruton’s tyrosine kinase (Btk) inhibitor ibrutinib in a phase II study of relapsed/refractory CLL patients, with ~90% of patients responding, and two complete responses (24). This combination is currently being assessed in a phase III trial of CLL patients (NCT02301156). Another phase III trial for this indication (NCT02612311) has been initiated involving a distinct combination regimen (see below) and ublituximab was placed on Reichart’s “Antibodies to watch in 2017” list (18).

On a final note, although the enhancement of ADCC with afucosylated mAbs cannot be disputed, a recent study utilizing mAbs to Rhesus D antigen (RhD) on erythrocytes indicated that afucosylated mAbs do not elicit greater ADCP, in comparison to a clear enhancement in ADCC (144). This led authors to conclude that the benefit of fucose removal may be restricted to cases where NK cells are known to be involved. How this relates to anti-CD20 mAbs is, therefore, of key interest, especially considering the predominant role of macrophages in this setting (see above).

### Fc Engineering

While glycosylation is a post-translational modification and, thus, difficult to precisely control, the IgG Fc backbone is readily amenable for mutation to create more efficacious molecules. Mutagenesis libraries have enabled the identification of IgG Fc variants that are aglycosylated but retain FcγR binding and effector functions similar to, or even exceeding that of, glycosylated IgG (145, 146). Extensive Fc backbone mutagenesis and an improved understanding of Fc–FcγR interactions has enabled the generation of mAbs with increased affinities for FcγRs and effector function (147). Multiple IgG mutations that increase binding for specific FcγR, both activatory or inhibitory, have been reported (148). 200-fold increased binding to FcγRIIB (but not FcγRIIA) was achieved through a Pro:Asp conversion at position 238 and generated IgG with increased agonistic capacity when applied to anti-CD137 mAb (149). Increased binding to FcγRIIIA alone, without impacting binding to FcγRI or the neonatal Fc receptor (FcRn) has also been reported using an anti-CD20 antibody (150). Increasing binding to activatory FcγRs but not FcγRIIB serves to increase the activatory:inhibitory (A:I) ratio (151), enabling greater effector cell activation. A 100-fold increase in ADCC was achieved using Fc mutation to increase FcγRIIIA binding (both high- and low-affinity alleles) and applied to several antibodies including rituximab (152). Fc mutations that improve binding to FcγRIIA selectively over FcγRIIB have also been reported, such as the G236A mutant, which resulted in improved macrophage phagocytosis (153). Furthermore, combination of this mutation with others can result in additive increases in ADCC and ADCP over the wild-type antibody (153).

AME-133v (now known as ocaratuzumab) is an example of an Fc-modified anti-CD20 mAb that is in clinical development for the treatment of B-cell malignancies (Table 1). AME-133v contains two mutations in its Fc region and elicits more efficient ADCC than rituximab with PBMCs from both FcγRIIIA VV158 and VF/FF158 patients (22). Moreover, 5/23 previously treated FL patients responded in a phase I/II clinical trial (22), suggesting potential efficacy. In separate *in vitro* studies, it was also indicated that ocaratuzumab is capable of mediating ADCC of CLL target cells at a greater level than rituximab and ofatumumab, and at a similar level to obinutuzumab (23).

As discussed above, several mutations are also able to promote hexamerization of IgG and elicit potent C1q binding leading to powerful CDC. Although (to the best of the authors’ knowledge) the effect of these mutations on FcγR binding has not been reported, there have been some reports that hexamer-enhanced mAb variants also have enhanced FcγR effector functions. To this end, de Jong et al. showed that variants (E345K and E430G) of the type II anti-CD20 mAb 11B8 mediated greater ADCC of Raji cells (82), and improvements in ADCC and ADCP were indicated in the setting of a modified immunomodulatory anti-OX40 mAb (154).

Notably, two situations whereby complement-optimized rather than Fc-optimized mAbs may be beneficial were highlighted in the aforementioned study by Lee et al. (107); reducing potential FcγR-mediated toxicity and FcγRIIB-mediated anti-CD20 mAb modulation, which has been suggested by us to be a rituximab resistance mechanism (73, 121). Finally, the authors speculated that complement-optimized mAb that work independently of FcγRs may be beneficial in the setting of unfavorable FcγR polymorphisms (107).

In addition to optimizing affinity of IgG for C1q and FcγR interaction, mutation strategies optimizing FcRn binding to improve serum IgG half-life has also been attempted to augment efficacy and reduce dosing frequency. Due to the pH-dependent binding of IgG to FcRn, improving the serum half-life of an IgG requires increased binding to FcRn at pH6 (allowing for greater FcRn binding in acidic endosomes) but unaltered FcRn binding at pH7.4 (thereby allowing release at the cell surface) (155). Numerous mutations have been reported to alter FcRn binding at pH6 (156). As an example, the M428L N434S double mutant on the IgG1 background of bevacizumab and cetuximab yielded increased FcRn binding (~10× fold for bevacizumab) and increased half-life in both human FcRn transgenic mice and cynomolgus monkeys (157). As far as we are aware, this technology has not been tested on anti-CD20 mAb. Given the shorter half-life of rituximab due to internalization, such an approach may be beneficial (73). A mAb targeting respiratory syncytial virus carrying the YTE triple mutant (M252Y/S254T/T256E) to increase FcRn binding at pH6.0 has been tested in humans and been reported to increase mAb half-life up to 100 days (158). Further optimization of Fc structure for optimal IgG half-life could enable the tailoring of IgG molecules to suit specific functions, including both therapeutic and also short-term uses such as labeling for imaging (159). Interestingly, enhanced FcRn binding through various Fc mutations has been combined with glycoengineering to generate low fucose anti-CD20 mAbs with increased serum half-life, FcγRIIIA binding, and ADCC (160).

## Isotype Selection and Engineering

All direct-targeting mAbs approved for use in oncology, including anti-CD20 mAbs, are of the IgG class (Table 1). However, it has been questioned whether IgG is the optimal therapeutic Ig class and whether efficacy could be improved by adopting other Ig classes. As expected, many of these proposals have used CD20 as their target of choice.

### IgA As an Alternative Ig Class

IgA is important in mucosal immunity (123) and, in contrast to IgG, it has only two isotypes (IgA1 and IgA2) (161). Much of the recent interest in using IgA as a therapeutic isotype has been in its potential to recruit the anti-tumor properties of neutrophils, which express the predominant (although not the only) receptor for IgA (FcαRI, CD89) (123). Crosslinking studies showed that CD89 signaling in neutrophils is efficient, and the use of bispecific mAb constructs (i.e., anti-CD20 × CD89) highlighted that stimulating the interaction between target antigen expressing tumor cells and CD89 on neutrophils efficiently induces cytotoxicity (162). A recent study also indicated that IgA mAbs targeting the melanoma antigen gp75, but not IgG1 or 3, mediated neutrophil ADCC *in vitro* (163). CD89 is also expressed by other myeloid cells including monocytes (and macrophages) (123). Therefore, considering the intricate involvement of macrophages in IgG mAb-mediated target cell depletion (see above), therapeutic IgA mAbs may be able to similarly engage and activate these cells when in sufficient number. However, when compared with IgG, IgA mAbs were limited in their ability to induce mononuclear cell ADCC, which is presumably due to the low percentage (10%) of monocyte effector cells within this cell population, and/or the presence of NK cells (20%) (109) that are not expected to engage IgA mAbs.

Anti-CD20 mAbs of the IgA class have been compared with IgG mAbs in various models. Surprisingly, anti-CD20 IgA2 was capable of mediating CD20 target cell depletion similar to IgG1 in an adoptive transfer model utilizing mice lacking CD89 (164). Pascal et al. also reported activity of IgA2 anti-CD20 in similar adoptive transfer models, although in this setting IgA2 was less effective than IgG1 anti-CD20 (165). Moreover, a different strategy was also employed, whereby DNA constructs encoding anti-CD20 IgG1 and IgA2 were vaccinated following tumor challenge to allow *in vivo* mAb synthesis and, thus, avoid difficulties in IgA purification (165). The survival of mice vaccinated with IgA2 and IgG1 constructs was similar, which is intriguing considering the absence of CD89 expression [as in Lohse et al. (164)]. However, a significantly increased activity of anti-CD20 IgA2 was reported in CD89 transgenic mice in comparison to wild-type mice (165), highlighting the potential for tumor cytotoxicity downstream of IgA interaction with cognate receptor-expressing effector cells *in vivo*.

In these anti-CD20 studies, it was shown that, as expected, IgA mAbs induced neutrophil-mediated cytotoxicity of both cell line and CLL targets to a greater extent than IgG, although (as expected) the converse was true for mononuclear cells (164). The same trend was observed with anti-HLA class II mAbs (109). Notably, however, IgA was able to recruit more immune cells than IgG in an *in vitro* imaging assay, in a CD89-dependent manner (165). Interestingly, these studies also showed that hIgA anti-CD20 mAbs were capable of inducing CDC of varying CD20^+^ target cells *in vitro* (164, 165). Although of interest, the relevance of this finding *in vivo* is unclear due to retained activity of anti-CD20 hIgA in C1q and C3 knockout mice (164). Despite differences in the kinetics of CDC mediated by IgG1 and IgA2 anti-CD20 being identified, as well as sensitivity to factors such as mAb (165) or serum concentration (164), the unexpected ability of IgA mAbs to induce CDC is nevertheless intriguing from a biological perspective, as IgA antibodies are not expected to engage C1q. Pascal et al. proposed an indirect mechanism for C1q binding downstream of anti-CD20 IgA (165) and recent studies have provided further evidence for a mechanism, now referred to as “accessory CDC,” which occurs in an Fc-independent, BCR-dependent fashion (166). Strikingly, mAbs with no expected CDC functions, namely anti-CD20 F(ab′)_2_ fragments or IgG4 mAbs with a complement-silencing mutation (K322A), were capable of inducing CDC of BCR^+^ cell lines. The emerging mechanism of such Fc-independent CDC is, therefore, reliant on clustering of the BCR by anti-CD20 mAbs, which favors indirect binding of C1q to surface IgM and subsequent CDC (166). The phenomenon may be limited to anti-CD20 mAbs, as no CDC was observed with IgA1 or IgA2 anti-HLA class II mAbs (109).

### IgGA Chimeras

Although IgA mAbs are clearly functional *in vivo*, it is not yet clear how IgA would replace IgG in clinical practice (164). Moreover, IgA molecules have disadvantageous attributes, such as a difficulty of purification and a shorter half-life in comparison to IgG (165). As described, IgA molecules are also not expected to stimulate NK cells, as evidenced by the absence of cytotoxicity observed with mononuclear cells in comparison to IgG (109, 164). For these reasons, there have been efforts to engineer novel mAbs containing the Fc regions of both IgG and IgA, with a view that the resulting molecule will harness the beneficial properties of both Ig classes. Kelton et al. grafted relevant regions of IgA into the Fc region of an anti-HER2 mAb to form a so-called “cross-isotype” IgGA mAb (167). The resulting IgGA mAbs were capable of binding to both FcαRI and FcγR, and induced neutrophil ADCC and macrophage ADCP of HER2^+^ targets similar to IgA molecules, and to a greater extent than parental IgG mAb. Next, as anti-HER2 mAbs did not elicit CDC, presumably due to the biology of the target, and similar to unmodified anti-EGFR (81, 82), anti-CD20 IgGA was generated. This was capable of inducing greater CDC of CD20^+^ targets in comparison to IgA, and greater CDC at lower concentrations than an IgG variant of the same mAb. However, anti-CD20 IgA did induce some CDC, although in contrast to Lohse et al. (164) this was to a lesser extent than anti-CD20 IgG. This is likely related to the “accessory CDC” mechanism (166) mentioned above.

Notably, the IgGA construct did not bind to FcγRIIIA or FcRn (167). As this would be predicted to negatively impact ADCC/ADCP and IgG recycling, respectively, the functionality of IgGA molecules *in vivo* would be interesting to assess. To this end, a recent study assessed the efficacy of a similar anti-CD20 IgGA molecule which had equivalent pharmacokinetics to anti-CD20 IgG1 (168). Anti-CD20 IgGA treatment of tumor bearing mice (transgenic for CD89 on CD14^+^ myeloid cells) led to an improved regression of tumors in comparison to IgG or IgA, in a CD89-dependent manner. Similarly, a peritoneal model was used to show that the activity of IgA or IgGA *in vivo* requires interaction with CD89 on monocytes/macrophages. However, a limitation of this model is that CD89 was restricted to CD14^+^ cells, with no neutrophil CD89 expression. It is also unclear whether the expression level of the CD89 is comparable to that seen in humans.

Alternatively, in contrast to the grafting used to produce the “cross-isotype” IgGA, Borrok et al. fused the entire CH2/hinge of IgA2 onto the C terminus of an anti-HER2 IgG1 to form a tandem IgG/IgA molecule (169). Similar to the IgGA, this molecule mediated enhanced neutrophil ADCC in comparison to both IgG and IgA2. However, by contrast, it was also capable of inducing NK-mediated ADCC due to retained FcγRIIIA binding (169), albeit lower than compared to afucosylated IgG1. Also in contrast to IgGA, tandem IgG/IgA also bound FcRn with a similar affinity to hIgG1 and had a correspondingly similar half-life to IgG1 *in vivo*, therefore overcoming one of the main limitations of IgA. This can be expected as the CH2–CH3 interface contains the IgG binding site for FcRn (170), and is maintained in this molecule. Finally, considering that this study focused on HER2 as a target, comparing anti-CD20 mAbs with a tandem IgG/IgA backbone with cross-isotype IgGA *in vivo* would be worthwhile to identify the most effective molecule.

In summary, IgA mAbs clearly engage various effector mechanisms and can exploit additional killing pathways (i.e., *via* CD89) compared to IgG. Although IgA in itself may not be able to replace IgG due to reasons of half-life and manufacturability, various chimeric fusions or combination regimens have been designed or suggested that combine the beneficial aspects of both IgG and IgA. It would be interesting to assess how these novel agents influence resistance mechanisms following anti-CD20 mAb therapy. For example, is trogocytosis (120) still induced by chimeric IgG/A molecules and how does this compare to wild-type IgA and G? As highlighted previously (109), an advantage of utilizing IgA mAbs is that interaction with the inhibitory FcγRIIB, known to limit effector cell activity (102), would not be expected. Similarly, IgA mAbs would not be expected to interact with FcγRIIB on the surface of malignant B-cells, thus limiting FcγRIIB-mediated modulation and removal of CD20/antibody complexes from the cell surface (73, 121). It would be interesting to assess how modulation compares with IgG/A chimeras, and whether further modifying these chimeras can reduce FcγRIIB binding to improve efficacy/limit resistance mechanisms.

### IgE As an Alternative Immunoglobulin Class for mAb Therapies

Further to IgA, the anti-tumor potential of IgE has recently been identified, leading to suggestions that IgE may be an alternative Ig class for mAb therapeutics. Although IgE is widely recognized as an Ig class implicated in allergy and responses to parasites, Nigro et al. have recently shown that IgE has a role in immune surveillance following tumor challenge (171). Various models were utilized to show that control of tumor growth was mediated in an IgE- and Fc epsilon receptor (FcεRI)-dependent manner, with an additional role for CD8^+^ T cells. Further to showing that tumors induce effective IgE responses that can limit tumor growth in a tumor challenge setting, this highlights that the FcεRI–IgE axis is worth considering in the setting of mAb therapy.

In the setting of anti-CD20, Teo et al. showed that an IgE mAb was capable of activating and inducing cytotoxicity, in an antigen-specific manner, through cells typically involved in allergic responses, namely mast cells or eosinophils derived from cord blood (172). The authors also highlighted the limitation of studies involving PBMCs as effectors (173), where the poor responses observed with IgE mAb are not considered in the absence or paucity of IgE effector cells. Moreover, a crucial concern was highlighted, in that there is a risk of anaphylaxis in the setting of a large circulating tumor burden following anti-CD20 IgE therapy (172). This prevented *in vivo* assessment of IgE anti-CD20 in this setting. It, therefore, needs to be considered how anti-CD20 IgE mAb therapies can be optimized to limit toxicity in patients. Nevertheless, an anti-MUC-1 mAb in a solid tumor model (4T1) was assessed (172). Although the efficacy of the mAb alone was limited, when utilizing a slightly different strategy to aid IgE and chemoattractant synthesis at the tumor site, tumor regression was observed. This highlights the importance of effector cell chemotaxis to the tumor site in the efficacy of anti-IgE mAb therapy.

### Alternative IgG Isotypes

In addition to belonging to the IgG class, all but two (Panitumumab, hIgG2 anti-EGFR; ibritumomab, mouse IgG1 anti-CD20) of the direct-targeting mAbs approved for cancer treatment also have a hIgG1 Fc region (Table 1). Therefore, further to altering the class of Ig, changing the isotype has been considered as an alternative to anti-CD20 hIgG1 therapy.

### IgG3 As an Alternative Isotype for mAb Therapies

Similar to IgG1, IgG3 is capable of effective Fc-dependent effector functions, such as CDC and ADCC (173). Indeed, IgG3 binds favorably to C1q (173) and broadly binds to FcγRs similar to IgG1 (174). There are numerous differences between IgG1 and 3, however. The latter bears an extremely long hinge region (IgG3—62 amino acids; IgG1—15) and is subject to extensive polymorphism (IgG3—13 allotypes; IgG1—4) (175). IgG3 also has a shorter half-life in comparison to other isotypes (176), an inability to bind protein A (173), and suffers from aggregation issues (177). In many ways, these mirror the disadvantages of IgA (see above). Despite this, some studies have suggested that IgG3 may be a more effective isotype for anti-CD20 mAbs, and have provided strategies to overcome the aforementioned limitations.

Rosner et al. showed that an IgG3 variant of rituximab (C2B8-IgG3) induces greater CDC than the corresponding IgG1 variant, with indications of superior sensitivity to low CD20 densities, such as in the case of CLL cells (177). However, ADCC and ADCP mediated by anti-CD20 IgG1 versus IgG3 were not compared in this study. This greater CDC capability of anti-CD20 IgG3 in comparison to IgG1 was also observed by Natsume et al., although they reported the converse for ADCC, with IgG1 being more effective (178). Similarly, although not in the context of anti-CD20, IgG1 was more capable of inducing ADCP of melanoma cells than IgG3 in a recent study (163) further suggesting that FcγR effector functions may not be improved in the setting of IgG3. A molecule comprising the advantageous regions of both IgG1 and IgG3 may, therefore, be beneficial. To this end, similar to the “cross-isotype” IgGA mAb described above, a domain switch variant of rituximab was generated by replacing the CH2/CH3 (Fc) of hIgG1 with same regions of IgG3. One particular mAb (1133) was identified that mediated superior CDC in comparison to hIgG1 and 3 and maintained a similar level of ADCC to hIgG1. Despite a potential benefit of the long hinge of IgG3 in introducing flexibility into the molecule (179), this finding suggests that the long hinge region of IgG3 is not responsible for the enhanced CDC (as 1133 contains the CH1 and hinge region of IgG1). Indeed, it has previously been suggested that a disulfide bond connecting the heavy chains, and not a hinge region *per se*, is required for CDC (179).

However, due to a loss in protein A binding, a known feature of IgG3 mAbs (173), and, therefore, concern about purification of the molecule on an industrial scale, the CH3 domain of mAb 1133 was further modified with increasing amounts of IgG1 sequence. This resulted in a molecule (113F) that was capable of binding to protein A and, importantly, maintained its superior CDC-inducing capabilities. Intriguingly, protein A and FcRn both bind to the CH2–CH3 interface of IgG (170), and the shorter half-life of IgG3 in comparison to hIgG1 has been shown to be caused by a single amino acid in this region (R435 in IgG3, H435 in other isotypes) that reduces the ability of IgG3 to compete with other isotypes of IgG for FcRn binding at pH 6 and, consequently, increases degradation (180). This is important to consider in the design of mAb therapeutics, but as 113F (in addition to binding to protein A) also contains the H435 site (178), poor pharmacokinetics should not be a limiting factor in this case. The polymorphic nature of IgG3 should nevertheless be considered if designing an IgG3 mAb therapy, as the IgG3 G3m(s,t) allotype contains H435 and has a correspondingly longer half-life (180).

Finally, it was shown that afucosylation improved the ADCC capacity of 113F but did not affect CDC, and that 113F resulted in more effective and prolonged B-cell depletion in a cynomolgus monkey model in comparison to IgG1 (178). This suggests that 113F may also be more effective than anti-CD20 hIgG1 in human patients.

In summary, studies with anti-CD20 mAbs have suggested that IgG3 mAbs may mediate more CDC in comparison to IgG1. However, this finding is inconsistent with distinct target antigens, indicating context-dependent rules. FcγR effector mechanisms of IgG3 may also be limited in comparison to IgG1 *in vivo*, despite having a half-life enhancing mutation (see above), as highlighted in a recent study (163), although whether this translates to CD20 mAbs is unknown. Nevertheless, chimeric IgG1/3 molecules have been developed to combine the effector mechanisms of both IgG1 and 3.

## Overcoming Resistance and the Immunosuppressive Microenvironment

The two decades of study of CD20 and its mAbs have provided us with a wealth of knowledge for how these reagents work and might be augmented. However, it has become increasingly clear that in addition to tumor intrinsic factors, such as expression level (181, 182), internalization (73), and trogocytosis (183), that tumor extrinsic factors associated with the tumor infiltrate are critical for determining mAb efficacy. A well-recognized hallmark of tumors is their ability to subvert and suppress the host immune system to facilitate their growth (184). Hematalogic malignancies exhibit this trend and this may contribute to the tumor resistance often seen with anti-CD20 therapies. For example, CLL cells have been reported to produce the anti-inflammatory cytokine IL-10, which is able to reduce macrophage cytokine production (185), and also to impact upon the gene expression of both CD4^+^ and CD8^+^ T cells and viability of CD4^+^ T cells through surface expression of Fas ligand (186, 187). In addition, certain B-cell subsets have also been reported to produce IL-10, which may contribute to an anti-inflammatory environment within lymphoid organs (188). Tumor-associated macrophages frequently display a pro-tumor phenotype characterized by reduced phagocytosis and production of angiogenic factors (189).

Anti-CD20 therapy has been shown to be highly effective at rapidly depleting CD20 expressing cells from the circulation (190–192). However, circulating B-cells constitute only approximately 2% of the total B-cell population, and thus the penetration and efficacy of anti-CD20 mAbs into lymphoid tissues is crucial to their effectiveness (193). Mouse and primate studies have indicated that increasingly large doses are needed to deplete B-cells from bone marrow, spleen, and lymph nodes (191, 194, 195). As many malignant B-cells reside in lymphoid organs, if they are not eradicated by anti-CD20 therapy, they can act as disease reservoirs enabling re-emergence of the tumor leading to relapse and progression (196). Although next-generation mAb such as obinutuzumab that have followed rituximab have improved depletion efficacy, it is clear that further improvements in treatment regimens are still required (16).

## Overcoming Resistance to Anti-CD20 Therapy through Combination

As described above, an immunosuppressive microenvironment is one mechanism known to reduce the efficacy of mAb treatment. As such, attempts to alter the tumor microenvironment to a more favorable, inflammatory state have been made. Agonists for toll-like receptors (TLRs), known to be important transducers of inflammatory signals in response to pathogen-associated molecular patterns such as LPS, are one group of molecules that have been tested. The synthetic oligodeoxynucleotide TLR agonist CpG, which activates TLR9, in combination with low dose radiotherapy has been reported to have a beneficial impact on B-cell lymphoma patients, inducing a T cell memory response in certain patients (197). Another TLR-9 agonist, 1018 ISS, has been combined with rituximab in follicular lymphoma and reported clinical response and tumor infiltration of CD8^+^ T cells and macrophages (198).

Another class of immunomodulatory molecules recently developed is STING agonists. These cyclic dinucleotides are sensed by cytosolic STING receptors (199). Normally involved in detection of DNA viruses, these agents can induce expression of IFN genes contributing to increased inflammation (199). *In vitro* and *in vivo* experiments using STING agonists have reported a phenotypic change of macrophages to a more inflammatory phenotype, increasing expression of activatory FcγRs crucial for antibody-mediated therapy (119). Accordingly, *in vivo* combination of STING ligands with anti-CD20 mAbs in a model of B-cell lymphoma overcame tumor-mediated immune suppression and resulted in curative treatments for 90% of mice (119).

An alternative immunomodulatory compound being assessed in combination with anti-CD20 mAb is lenalidomide. Lenalidomide is thought to act both through inducing tumor cell death and altering the tumor microenvironment and is approved for use in multiple myeloma (200). Lenalidomide combined with anti-CD20 mAb resulted in a significantly greater overall and complete response rates versus lenalidomide alone in a meta-analysis of refractory/relapsed CLL patients (201). Interestingly, lenalidomide plus anti-CD20 mAb achieved similar complete response rates to those seen with ibrutinib plus rituximab (see below) (202). Lenalidomide plus rituximab has also reported high response rates in untreated indolent NHL (203). The mechanistic basis for these effects is not yet fully resolved.

An alternative means of achieving immune conversion is by combining anti-CD20 mAbs with the so-called immunomodulatory antibodies. These antibodies differ from direct-targeting mAb in that they bind to cells of the immune system (rather than the tumor target) with the aim of activating or de-repressing them to elicit T cell responses. These mAb have achieved remarkable success in the last few years in treating certain patients with melanoma and lung cancer (6). The possibility of combining these agents with direct-targeting anti-CD20 mAbs has been proposed and tested in clinical trials. One such study combined the anti-programed cell death-1 (PD-1) antibody pidilizumab with rituximab in the treatment of relapsed/refractory follicular lymphoma (204). Albeit for a small sample group, this study reported an increased complete response rate of 52% as compared to only 11% in patients receiving rituximab monotherapy. Nivolumab, another anti-PD-1 antibody, has already been approved for use in refractory Hodgkin’s lymphoma after stem-cell transplant (205).

Following a phase I trial finding, ipilimumab was well tolerated in NHL and increased T cell proliferation. A combination trial involving rituximab and the anti-CTLA-4 antibody ipilimumab is ongoing (206).

Other strategies for improving anti-CD20 therapy aim to address the results of tumor-mediated immune suppression, rather than reverse them *per se*. In our own work, we have attempted to counter the above described FcγRIIB-mediated internalization and inhibitory signaling which decreases CD20 therapy efficacy. This has been achieved through the use of an antagonistic anti-FcγRIIB antibody that prevents the *cis* binding of anti-CD20 antibody to FcγRIIB on the same cell, preventing internalization (131). Furthermore, this effect was also shown for combination of obinutuzumab and alemtuzumab with anti-FcγRIIB, suggesting a more general mechanism for reducing antibody internalization and increasing therapeutic efficacy. This has led to the initiation of a clinical trial for combining rituximab with anti-FcγRIIB in FcγRIIB^+^ cell malignancies (NCT02933320).

In addition to these immune-related interventions detailed above, recent years have also seen a rapid increase in drugs targeted at specific molecules thought to be involved in malignancy. In many cases, these have been combined with anti-CD20 mAbs for the treatment of B-cell malignancies. One such drug, ibrutinib (Ibruvica), an irreversible inhibitor of Btk has been approved for the treatment of relapsed/refractory CLL and several NHLs owing to high response rates and increased survival (207). Ibrutinib has been combined with anti-CD20 chemoimmunotherapy and yielded increased response rates in relapsed/refractory CLL over chemoimmunotherapy alone (202, 208). Ibrutinib has also been combined with anti-CD20 mAb in, among others, DLBCL and MCL and has achieved high response rates (209, 210). Further trials are ongoing combining ibrutinib with chemoimmunotherapy in various disease settings (211). Despite the apparent efficacy of this combination, ibrutinib has been reported to decrease antibody-induced cell-mediated effector mechanisms both *in vitro* and in cells from patients taking ibrutinib (212). This highlights the importance of considering drug combination mechanisms of action and appropriate dosing schedules to get the maximum benefit for patients.

Another small molecule inhibitor, idelalisib (Zydelig), approved for relapsed/refractory CLL and FL therapy is targeted at the delta isoform of the lipid kinase phosphoinositide-3-kinase (PI3Kδ) (213, 214). This isoform is preferentially expressed in leukocytes and expressed in malignant B-cells (215, 216). Targeting of PI3Kδ has shown to be effective in the treatment of B-cell malignancies, although toxicity issues have prevented idelalisib from becoming a front line therapy (217, 218). Combination of idelalisib and rituximab was found to be superior to idelalisib alone in relapsed/refractory CLL, and addition of idelalisib to bendamustine–rituximab therapy for CLL patients with a poor prognosis has shown to improve progression-free survival (219, 220). Idelalisib has also shown efficacy in several NHLs as monotherapy and in combination with rituximab and bendamustine (221, 222). Recent work from our group has revealed the pro-apoptotic BH3-only protein Bim to be key to the *in vivo* therapeutic mechanism of PI3Kδ inhibition. Addition of a PI3Kδ inhibitor to anti-CD20 mAb therapy reduced the accumulation of leukemia cells in the Eμ-Tcl1 transgenic mouse model, and also improved survival compared to anti-CD20 mAb or PI3Kδ inhibitor alone, in a Bim-dependent manner (223). Furthermore, combination of a PI3Kδ inhibitor with a BCL-2 inhibitor was more effective than either agent alone, reducing leukemic burden by 95% (223).

Venetoclax (Venclexta) is another small molecule inhibitor that targets BCL-2 and is approved for the treatment of relapsed/refractory CLL with 17p chromosomal deletions, based on high response rates in heavily pretreated patients (224, 225). This molecule has also been trialed in combination with rituximab in relapsed/refractory CLL, with high response levels reported (86% overall response rate) (226). Trials combining venetoclax with obinutuzumab are also underway, with preliminary data suggesting that it is highly efficacious in relapsed/refractory and untreated CLL in elderly patients (227, 228). Importantly, venetoclax has been reported to be efficacious in CLL patients who have failed previous kinase inhibitor therapy, such as ibrutinib or idelalisib (229). Another anti-BCL-2 drug, the antisense oligonucleotide Oblimersen sodium, has been tested in combination with rituximab and found to be beneficial in patients with relapsed/refractory NHL (230).

Although segregated in this review by mechanism, combinations of multiple drugs with differing mechanisms of action are being examined alongside anti-CD20 therapy. For example, TG Therapeutics are currently recruiting patients with relapsed/refractory CLL to a trial combining ublituximab (a glycoengineered anti-CD20 antibody) with TGR-1202 (a PI3Kδ inhibitor) and pembrolizumab (anti-PD-1 antibody). Whether such an approach is efficacious or indeed viable in terms of health economics remains to be seen.

## Bispecific Antibodies (bsAbs)

A further therapeutic approach that is currently being trialed in the clinic is the use of bsAbs. Multiple technologies have been developed for producing bsAbs, incorporating additional Fab domains in various positions and with altered Fc backbone engineering to ensure appropriate heavy chain pairing (231). A bsAb targeting CD19 and CD3 has already achieved approval for relapsed/refractory acute lymphoblastic leukemia (232). An anti-CD20/CD22 bsAb has shown enhanced preclinical activity over the combination of the two parental antibodies, inducing greater apoptosis *in vitro* and improved overall survival and tumor shrinkage *in vivo* (233). Combination of two anti-CD20 mAbs (a type I and a type II) into a tetravalent bsAb produced a molecule that induced enhanced direct cell death over the combination of parental Abs and retained equivalent CDC (234). Furthermore, this molecule had a more potent anti-tumor activity than the combined parental antibodies *in vivo*.

Attempts to increase engagement of the target cell with effector cells using bsAbs have also been made. One example is a CD20(2) × FcγRIIIA tribody that binds target CD20 and effector FcγRIIIA, irrespective of the V/F158 polymorphism. This construct was superior to rituximab in terms of cell line and patient lymphoma cell lysis, NK-mediated tumor cell killing, and also B-cell depletion in whole blood, and functioned to deplete human B-cells in a mouse model reconstituted with a humanized hematopoietic system (235). A CD20/CD3 bsAb tested in multiple *in vivo* models appeared to act primarily through CD3 expressing cells, rather than the antibody Fc region of this bispecific humanized IgG (236). Some of these bsAbs, such as the CD20/CD3 molecules REGN1979 (237) and FBTA05 (238), have entered clinical trials for B-cell lymphoma. Despite the termination of the clinical trial for FBTA05, this antibody has been used on compassionate grounds in children with B-cell malignancies refractory to conventional therapy, with some positive results (239).

## CD20 mAb in Autoimmune Settings

In addition to the treatment of B-cell malignancies, many of the same therapeutic principles learnt from the study of anti-CD20 mAb can be applied to other disease settings, namely autoimmune disease. The rationale for B-cell depletion in autoimmune diseases such as rheumatoid arthritis (RA) is based on the (albeit incompletely understood) role of these cells in disease pathogenesis, namely differentiation into autoantibody-secreting plasma cells and antigen presentation to T cells, and the consequent expectation that their depletion will restore self-tolerance, as discussed in depth elsewhere (240). Nevertheless, it was shown in a double-blind randomized control trial that treating RA patients with rituximab resulted in both prolonged B-cell depletion and significant improvements in symptoms in comparison to methotrexate-treated patients (241). Moreover, a combination of rituximab and methotrexate increased the percentage of patients with improvements in symptoms at 48 weeks post-treatment (241). As a consequence of this (and other studies), rituximab is now FDA-approved for the treatment of RA, as well as the anti-neutrophil cytoplasmic antibody (ANCA)-associated vasculitides (AAV), Wegener’s Granulomatosis and Microscopic Polyangiitis (https://www.fda.gov/Drugs/DrugSafety/ucm109106.htm). However, contrary to indications of efficacy (242), rituximab showed no significant clinical benefit over control arms in randomized clinical trials of both extrarenal (243) and renal (lupus nephritis) (244) systemic lupus erythematosus (SLE) patients. Nevertheless, it has been estimated that rituximab is used off-label in approximately 0.5–1.5% of SLE patients in Europe, seemingly as a last resort in patients with worse disease (245).

As may be expected, a requirement for FcγRs in the mechanism of action of rituximab in autoimmune disease (as for B-cell malignancies) has been indicated in studies such as by Quartuccio et al., whereby clinical responses of RA patients were significantly greater at 6 months post-rituximab in FcγRIIIA V/V patients (246). It is noteworthy that the depletion of B-cells by rituximab may be variable (between patients) and incomplete in autoimmune disease. In the setting of RA, for example, a sensitive flow cytometry technique was used to detect remaining B-cells, and patients with complete depletion of B-cells after a single rituximab infusion had favorable clinical responses in comparison to patients with partial depletion (247). Similarly, when the same methodology was applied to SLE, all patients with complete B-cell depletion had a clinical response to rituximab, which contrasts to patients with incomplete B-cell depletion (248). Intriguingly, a significantly lower depletion of B-cells from SLE patients was observed in comparison to B-cells from RA patients or healthy donors when treated with anti-CD20 mAb in whole blood assays (249).

Several mechanisms may help to explain the variable and/or incomplete B-cell depletion observed with rituximab in autoimmune disease. This may be linked to levels of B-cell-activating factor (BAFF), which is known to increase in RA patients treated with rituximab in periods of B-cell depletion (240). Indeed, a recent retrospective study analyzed two cohorts of AAV patients and showed that a single nucleotide polymorphism in BAFF (TNFSF13B) was associated with responses to rituximab treatment (250). Although the authors of this study conceded that further mechanistic studies are required, this indicates that responses to B-cell depletion may be predicted in advance of rituximab treatment in the future (similar to FcγR polymorphisms and degree of B-cell depletion mentioned above), and patients given alternative therapies instead. Modulation of FcγRIIB/rituximab complexes may, as for malignant B-cells (73, 121), also be a relevant resistance mechanism in the setting of autoimmune B-cells, as indicated in *in vitro* studies (249) (see below). Finally, results from animal models of SLE have suggested that inefficient depletion in this disease may be due to the presence of autoantibody ICs (251). Recent studies employing chronic viral infection models, also characterized by excessive ICs, have lent support to the hypothesis that high concentrations of ICs may inhibit antibody effector mechanisms (252, 253). Both of these studies utilized anti-CD20 mAb and showed that chronically infected mice were incapable of depleting CD20^+^ cells (252, 253). This suggests that high levels of circulating ICs should be a concern in setting of anti-CD20 therapy and may result in inefficient B-cell depletion in patients.

Nevertheless, considering such indications of incomplete B-cell depletion using rituximab in autoimmune disease, one fundamental question is how the depletion of B-cells can be improved in the setting of autoimmune disease. Employing next-generation mAbs is an option. To this end, although a non-glycoengineered type II anti-CD20 mAb induced greater depletion of B-cells in comparison to rituximab in a whole blood assay (249), suggesting a role for the type II nature of the mAb rather than a change in glycosylation, depletion was further increased with the glycomodified (afucosylated) type II mAb obinutuzumab (17). The greater depletion mediated by type II anti-CD20 corresponded to less internalization from the surface of B-cells from healthy donors and RA/SLE patients (249). In the setting of SLE, B-cell depletion by rituximab correlated with the level of surface accessible CD20, and the difference between B-cell cytotoxicity mediated by type I versus type II anti-CD20 mAb correlated with degree of internalization (249). Internalization mediated by type I anti-CD20 could be partially inhibited by use of blocking anti-FcγRIIB mAb (249). It can, therefore, be hypothesized that a combination of rituximab with an anti-FcγRIIB mAb will increase the efficiency of autoimmune B-cell depletion, for reasons including blockade of such FcγRIIB-mediated modulation, or FcγRIIB-mediated inhibition of activatory signaling on effector cells (102). Further still, alternative anti-CD20 mAbs have also been/are being developed for the treatment of other autoimmune diseases, namely the humanized mAb veltuzumab for the treatment of ITP (in addition to CLL/NHL) (20), which has a single amino acid change in the complementary determining region (CDR) 3 V_H_ in comparison to rituximab, and framework regions/Fc domains from the anti-CD22 mAb epratuzumab (21); and ocrelizumab for the treatment of multiple sclerosis (MS) (Table 1). Notably, ocrelizumab was recently shown to significantly decrease disease progression in a phase III trial of primary progressive MS when compared with placebo (19) and was successful in two other trials (18), leading to its FDA approval. Alternatively, the glycoengineered anti-CD20 mAb ublituximab (Table 1) is also in clinical trials for the treatment of MS (25) for reasons of increased ADCC/potency (see above). Nevertheless, as with RA (241), the clinical benefit observed following B-cell depletion with anti-CD20 in MS further emphasizes a role of these cells in autoimmune disease pathogenesis (19).

A final factor to consider is the existence of serological evidence of autoimmunity that can precede the development of overt disease by years, as reviewed elsewhere (254, 255). It has, therefore, been questioned whether the development of autoimmune disease can be prevented/delayed. Studies such as PRAIRI (256) have, therefore, tested this, by infusing autoantibody-positive patients that do not yet have overt RA with a single infusion of rituximab (1,000 mg) and prospectively monitoring for disease onset versus placebo controls. The early results indicate that this strategy is able to delay disease onset (256).

## Conclusion and Summary

Anti-CD20 mAbs have now been with us as approved clinical reagents for 20 years. As highlighted in Figure 1, their development and study has fostered a large amount of our current knowledge of therapeutic mAb mechanisms of action and what makes an effective therapeutic target and mAb. In the next 5 years, an increasing number of combination strategies will be investigated in order to improve on the current levels of success. Coupled to this will be an increasing number of new mAb formats, aiming to take advantage of the knowledge gained to date. One important aspect of this development will be an in depth understanding of the disease microenvironment in each case. For example, to improve responses in CLL may not require the same developments as required for NHL and similarly the specific pathologies relating to RA, SLE, and MS may not involve similar solutions.

More widely, we can expect the learnings gleaned from the study of CD20 antibodies will flow into developments for other mAb specificities; particularly where target cell deletion is required. So, in answer to the question “What have we learnt from targeting CD20 and where are we going?” the response should be “a huge amount” and “to an era of combination and advanced antibody engineering leading to improved responses for patients.”

## Author Contributions

MM and RS wrote the manuscript and both contributed equally. MC edited and wrote the manuscript.

## Conflict of Interest Statement

MC is a retained consultant for Bioinvent and has performed educational and advisory roles for Baxalta and GLG. He has received research funding from Roche, Gilead, and GSK. MM and RS both receive BBSRC iCASE studentships and are partly funded by Roche and Promega, respectively. The reviewer MI and handling editor declared their shared affiliation.
